# Retained drains causing a bronchoperitoneal fistula: a case report

**DOI:** 10.1186/1752-1947-5-185

**Published:** 2011-05-14

**Authors:** Catherine Pesce, Samuel M Galvagno, David T Efron, Alicia A Kieninger, Kent Stevens

**Affiliations:** 1 Johns Hopkins Hospital Department of Surgery, Baltimore MD, USA; 2 Johns Hopkins Hospital, Department of Anesthesiology and Critical Care Medicine, Division of Adult Critical Care Medicine, Baltimore, MD, USA; 3 Johns Hopkins Hospital, Department of Surgery, Baltimore, MD, USA; 4 Washington University School of Medicine, Department of Acute and Critical Care Surgery, Barnes Jewish Hospital, St Louis, MO, USA

## Abstract

**Abstract:**

**Introduction**

Bronchoperitoneal fistulas are extremely rare. We present a case where retained surgical drains from a previous surgery resulted in erosion and fistula formation. This condition required an extensive surgical procedure and advanced ventilator techniques.

**Case presentation**

A 24-year-old African-American man presented to our Emergency Department with a one-week history of fever, dyspnea, cough, and abdominal pain. A computed tomography scan of his chest and abdomen revealed bilateral lower lobe pneumonia and two retained Jackson-Pratt drains in the right upper quadrant. He was taken to the operating room for drain removal, a right hemicolectomy, debridement of a duodenal injury, a Roux-en-y duodenojejunostomy, and an end ileostomy. He subsequently became increasing hypoxemic in the intensive care unit and a bronchoperitoneal fistula was diagnosed. He required high-frequency oscillatory ventilation followed by lung isolation, and was successfully resuscitated using these techniques.

**Conclusion:**

To the best of our knowledge, this is the first known case report of a bronchoperitoneal fistula caused by retained surgical drains. This is also the first known report that details successful management of this condition with advanced ventilatory techniques. This case highlights the importance of follow-up for trauma patients since retained surgical drains have the potential to cause life-threatening complications. When faced with this condition, clinicians should be aware of advanced ventilatory methods that can be employed in the intensive care unit. In this case, these techniques proved to be life-saving.

## Introduction

The formation of a fistula between the bronchus and peritoneal cavity is exceedingly rare. Most causes are due to subphrenic abscess or iatrogenic biliary procedures causing diaphragmatic rupture [1-4]. We present a case where retained Jackson-Pratt drains from a previous surgery precipitated diaphragmatic erosion and resultant fistula formation.

## Case presentation

**Figure 1:**
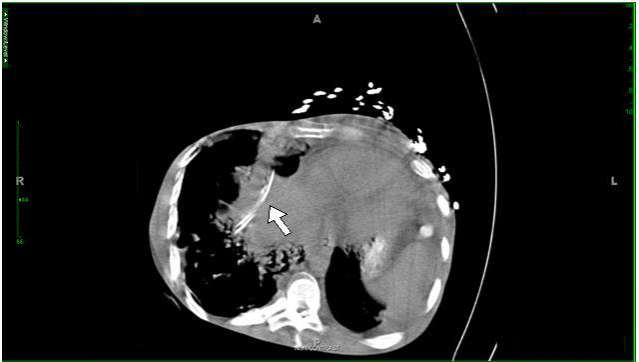
**A retained Jackson-Pratt drain resting over the dome of the liver and traversing the right posterior hepatic space and transverse colon**.

A 24-year-old African-American man presented to our Emergency Department with a one-week history of fever, dyspnea, cough with brownish phlegm, and abdominal pain. A computed tomography (CT) scan of the chest and abdomen revealed bilateral lower lobe pneumonia and two retained Jackson-Pratt drains in the right upper quadrant with a small amount of subcutaneous emphysema and stranding adjacent to one of the drain tips (Figure 1). In addition to the presence of pneumoperitoneum, one drain was resting over the dome of the liver and traversing the transverse colon, while the other drain was near the right flank and distally traversing the second portion of the duodenum. Neither drain could be appreciated by palpation or inspection along the external body surface.

His history was significant for an exploratory laparotomy, right nephrectomy, and diaphragm repair for a gunshot wound sustained to the right lower back and abdomen eight years previously. The entrance wound was via the diaphragm into the abdomen, with the bullet traversing segments eight and five of the liver and then into the retroperitoneum. There was no significant bleeding from the right lobe liver wound, but there was a large zone 2 retroperitoneal hematoma on the right. The mid-portion of the parenchyma of the right kidney had been essentially obliterated, and the pelvis of the right kidney was involved; the right kidney was therefore resected. The diaphragm injury was repaired with a running #0 Prolene suture and then two separate 3/16 inch round silastic drains were placed, one over the dome of the liver, the second into the subhepatic pararenal space. Our patient was discharged home with the drains left in place for large output drainage. He stated that a non-medically trained acquaintance cut the surgical drains at the skin several months after he had been sent home. He was taken to the operating room for drain removal, right hemicolectomy, debridement of duodenal injury, Roux-en-y duodenojejunostomy, and end ileostomy. On exploration, one drain was found eroding into the duodenum causing a chronic duodenotomy that had previously communicated with the sinus tract on our patient's anterior abdominal wall. The defect was in the lateral wall on the anti-mesenteric side of the c-loop of the duodenum. The duodenum was inspected carefully intra-luminally and the ampulla was identified clearly. It was therefore felt safe to debride the edges and perform a side-to-side duodenojejunostomy with a Roux-en-y limb. A 10-mm Jackson-Pratt drain was left near the anastomosis. There were no signs of anastomotic leakage post-operatively. During the case, bubbling of air was noticed near the dome of the liver, and while under anesthesia, our patient sporadically became hypoxemic despite ventilation with 100% oxygen. However, no obvious diaphragmatic injury could be identified intra-operatively. He was admitted to the surgical ICU for further post-operative care.

**Figure 2:**
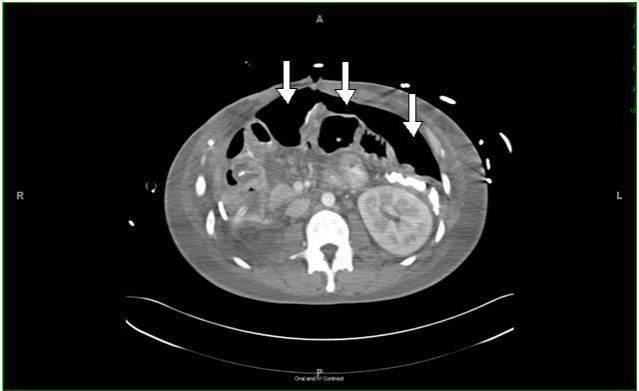
**Post-operative computed tomography (CT) scan demonstrating a large volume pneumoperitoneum due to bronchoperitoneal fistula**.

Over the course of the next 48 hours, he remained hypotensive, hypoxemic, acidotic, and increasingly dys-synchronous with the ventilator. Both peak airway pressures (38 to 42 cm H_2_O) and plateau pressures (36 to 38 cm H_2_O) remained elevated and the chest radiograph showed bilateral patchy infiltrates consistent with acute respiratory distress syndrome (ARDS), and no pneumothorax. Multiple ventilation modes were attempted, including all available pressure-limiting modes as well as bi-level ventilation and airways pressure release ventilation (APRV) to improve oxygenation and attenuate the high airways pressures. During conventional ventilation, approximately 60 mL per breath of air leak was observed in a right upper quadrant Jackson-Pratt drain, and a persistent air leak of greater than 100 mL/mL breath was found in the right-sided thoracostomy tube that had been placed intra-operatively. A total of four thoracostomy tubes were eventually placed: two at each apex and two at each base. Brochoalveolar lavage (BAL) specimens recovered from the right middle and right lower lobes grew pan-sensitive *Escherichia coli*. During serial bronchoscopies, no laceration or evidence of fistula could be identified. A CT scan was obtained which showed a large volume pneumoperitoneum, and an old drain exit site near the right upper quadrant was leaking air (Figure 2). A bronchoperitoneal fistula had formed from drain erosion into the diaphragm, connecting the peritoneal and pleural cavities. Due to his severely compromised lung function and progressive hypoxemia (partial pressure of oxygen in the blood (PaO_2_) of 54 on 100% fraction of inspired oxygen (FiO_2_)), he was sedated, paralyzed, and high-frequency oscillatory ventilation (HFOV) was initiated with a frequency of 5 Hz, FiO_2 _of 100%, mean airway pressure (mPaw) of 44 cm H_2_O, and oscillation pressure amplitude (ΔP) of 90 cm H_2_O. Over the course of four days, the frequency was gradually increased to 10 Hz, the FiO_2 _was weaned to 0.4, the mPaw was decreased to 26 cm H_2_O, and the ΔP was decreased to 30 cm H_2_O. As his oxygenation and hemodynamic status improved, he was removed from HFOV and a left-sided double-lumen tube was inserted to provide differential lung ventilation. After two days of differential ventilation with the right lung receiving a tidal volume of 150 mL, FiO_2 _of 40%, PEEP of 0 cm H_2_O, with peak inspiratory pressures of 14 to 18 cm H_2_O, the air leaks from both the abdominal drain and chest tubes were found to have stopped completely. Our patient received a tracheostomy, mechanical ventilation was eventually discontinued, and the chest tubes were removed. He was discharged home and had a documented full functional recovery with an osteomy takedown completed one year later.

## Discussion

Bronchoperiotoneal fistulas are rare phenomena [5]. The connection between the bronchi and peritoneal cavity usually forms in a cephalad direction, however one report has cited a lung abscess as an origin [6]. The most common etiologies include subphrenic abscess and iatrogenic percutaneous biliary procedures causing diaphragmatic rupture; however, thoraco-abdominal trauma, malignancy, and suppurative biliary tract obstruction have also been reported [2].

To the best of our knowledge, this is the first report of retained drains causing diaphragm erosion and fistula formation. The fistula was recognized in the post-operative period when our patient became increasingly septic with increasing oxygen and vasopressor requirements. The combination of larger-than-expected post-surgical pneumoperitoneum on abdominal CT, and an air leak from an old site, ultimately led to the diagnosis.

In our patient, healing of the fistula was accomplished by employing the same strategy used to treat bronchopleural fistulas. Both HFOV and differential ventilation have been used as novel methods for managing ventilation in patients with bronchopleural fistulas [7-10]. In this case, HFOV was initially utilized to improve oxygenation, limit further barotrauma, decrease air leak, and eventually, lower peak airway pressures. Limited data on the use of HFOV indicate that this technique has been used successfully in the past for bronchopleural fistulas [8,9,11-13]. Purported beneficial mechanisms include a decreased risk of air trapping, less distension of airspaces, and better lung protection in patients with acute lung injury [11,12]. In patients with a bronchopleural fistula and acute lung injury, the fistula provides a low impedance pathway to airflow, resulting in a large air leak [12]. If ventilatory frequency is increased, the contribution of compliance to the impedance pathway decreases, thereby attenuating the degree of air leak [12]. Airflow was later limited through the tract via a double lumen endotracheal tube allowing for tract closure. This brief period of differential lung ventilation likely helped decrease airway pressures on the right, allowing the diaphragmatic and bronchopleural defects to close. With more severe bronchoperitoneal fistulas and larger diaphragmatic defects, treatment may require debridement of bronchopulmonary tissue, repair of diaphragmatic perforations, drainage of any subphrenic infected space, and antibiotics. Cases of bronchoperitoneal fistulas due to subphrenic abscess often require surgery. Treatment may include debridement of bronchopulmonary tissue, repair of diaphragmatic perforations, drainage of subphrenic infected space and adequate antibiotics. In cases of bronchobiliary fistulas due to iatrogenic biliary trauma, one of the primary goals of treatment includes relief of biliary obstruction if present. Early recognition and proper surgical management are necessary to prevent morbidity and mortality.

## Conclusion

Bronchoperitoneal fistulas, while rare, can be life-threatening. This case highlights the importance of patient education and follow-up for traumatically injured patients. Bronchoperitoneal fistulas may also be difficult to diagnose, and when identified, the patient may present in extremis. We describe the use of advanced ventilatory techniques that may be employed to allow the fistula to heal.

## Consent

Written informed consent was obtained from the patient for publication of this case report and any accompanying images. A copy of the written consent is available for review by the Editor-in-Chief of this journal.

## Competing interests

The authors declare that they have no competing interests.

## Authors' contributions

CP and SG analyzed and interpreted the data from our patient regarding the surgical procedure, history, and intensive care unit course. All authors read and approved the final manuscript.
